# Tubulointerstitial Nephritis and Uveitis: A Case Report

**DOI:** 10.7759/cureus.56405

**Published:** 2024-03-18

**Authors:** Michael Mira, Yuriy Khanin, Miroslav Sekulic, David Jordanovski

**Affiliations:** 1 Internal Medicine, Overlook Medical Center, Summit, USA; 2 Nephrology, Overlook Medical Center, Summit, USA; 3 Pathology, Columbia University, New York, USA

**Keywords:** acute kidney injury, acute kidney disease, tubulointerstitial nephritis with uveitis, tubulointerstitial nephritis and uveitis syndrome, tinu

## Abstract

Tubulointerstitial nephritis and uveitis (TINU) is a rare disease of unknown pathogenesis that is characterized by tubulointerstitial nephritis and uveitis. Currently, there are over 250 reported cases of TINU syndrome. TINU syndrome typically presents with mild uveitis and nephritis that is self-limited; however, in this case, the symptoms were severe making it different from previous case reports. We present a case of a 29-year-old female with a history of cytomegalovirus (CMV) with a recent diagnosis of bilateral uveitis who was admitted for worsening systemic symptoms. Laboratory testing revealed acute renal insufficiency along with hematuria and proteinuria. A kidney biopsy revealed tubulointerstitial nephritis, and the patient was initiated on corticosteroids for the diagnosis of TINU. The patient’s renal function recovered to baseline after a prolonged three-month course of systemic steroids but had a recurrence of her uveitis with steroid taper requiring initiation of steroid-sparing therapies. TINU syndrome should be considered in patients presenting with uveitis and renal dysfunction. Prompt diagnosis is necessary to preserve renal function with corticosteroids. The prognosis for patients with TINU is variable, with a frequently recurring and relapsing course. More research is needed to determine the optimal treatment.

## Introduction

Tubulointerstitial nephritis and uveitis (TINU) syndrome is a rare disease that is characterized by acute tubulointerstitial nephritis and preceded, accompanied, or followed by uveitis. Having been first described by Dobrin et al. [1] in 1975, now more than 250 cases have been reported around the world [2]. Most cases are adolescents and young women [3]. We present a case of a 29-year-old female who was admitted with one month of low-grade fevers, uveitis, and kidney injury ultimately culminating in a kidney biopsy revealing a diagnosis of TINU syndrome.

## Case presentation

A 29-year-old woman with a medical history of cytomegalovirus (CMV) infection 10 years ago developed mouth ulcers, joint pain and one month of low-grade temperatures, diffuse muscle aches, and an episode of abdominal pain that prompted her to go to the emergency room (ER). At that time, complete blood count and complete metabolic panel were unremarkable and CT abdomen and pelvis did not reveal any pathology. The pain was self-limiting. A few weeks later, she developed photosensitivity. She was evaluated by her ophthalmologist, revealing bilateral uveitis for which she was treated with prednisone eye drops. Shortly, thereafter, she developed a constellation of symptoms including fevers, chills, mouth sores, malaise, and arthralgias. Ultimately, she was started on amoxicillin for three days for a possible urinary tract infection that was diagnosed by her primary care doctor, but symptoms persisted, and she presented to the hospital for further evaluation.

Vital signs and physical examination were unremarkable on presentation. Laboratory analysis revealed WBC of 11.99 x10^9^/L, hemoglobin of 9.3 g/dL, serum creatinine of 1.75 mg/dL, and blood urea nitrogen (BUN) of 13 mg/dL. Erythrocyte sedimentation rate (ESR) was elevated to 66 mm/hr, and C-reactive protein was raised to 63.3 mg/dL. Urine protein excretion collected 24 hours was 888.8 mg/m^2^, and the microalbumin-creatinine ratio was 179.3 mg/g. Serum protein electrophoresis showed normal total protein and albumin with increased alpha 1 globulin at 0.5 g/dL. Free light chain testing showed an increased kappa chain of 3.99 mg/dL and normal lambda with an elevated kappa/lambda ratio of 1.74. Immunologic testing revealed elevated C4 complement at 66 mg/dL, and anti-nuclear antibodies (ANA) were positive (1:80 titer). In addition, anti-neutrophil cytoplasmic antibodies (ANCA), rheumatoid factor, cyclic citrullinated peptide (CCP) cardiolipin, human leukocyte antigen (HLA)-B27 haplotype, and direct Coombs tests were negative. The level of angiotensin-converting enzyme (ACE) was normal. Serological tests for Lyme disease, HIV 1 and 2, hepatitis C, tuberculosis, and syphilis were negative.

**Figure 1:**
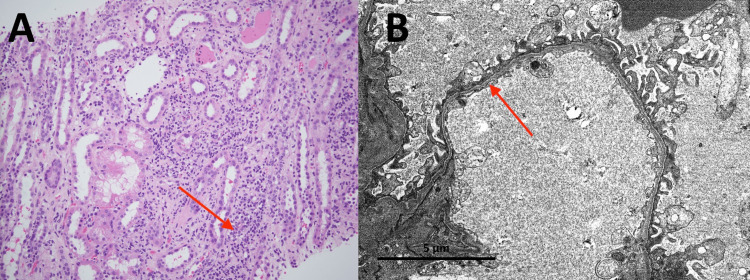
Kidney biopsy A. Light microscopy showed tubulointerstitial inflammation (red arrow) composed of mononuclear cells and several eosinophils, neutrophils, and plasma cells. B. Electron microscopy revealed diffuse attenuation of the glomerular basement membranes (red arrow) without splitting, fraying, or a "basket-weave" appearance of the lamina densa.

A kidney biopsy was performed, and histopathology revealed acute tubulointerstitial nephritis along with diffuse thinning of the glomerular basement membrane with a mean diameter of 160 nm consistent with thin basement membrane disease (Figures 1A, 1B). The inflammatory cells were primarily neutrophils. Due to the findings of predominantly neutrophil on histopathology, there was concern for pyelonephritis. An extensive workup was performed with urinalysis, urine culture, and CT abdomen without contrast did not reveal obvious signs of infection. 

The provisional diagnosis of TINU syndrome was made, and she was placed on oral prednisone 60 mg daily tapered over three months, along with prednisone eyedrops. On follow-up, her renal function slowly improved with a serum creatinine of 1.19 mg/dL after completion of the steroid taper; however, she had a recurrence of uveitis requiring initiation of steroid-sparing therapy with Methotrexate 2.5 mg weekly. Due to mood disturbances and abnormal liver function enzymes, she was transitioned to mycophenolate mofetil. Unfortunately, her uveitis persisted and she is now planned for an adalimumab infusion.

## Discussion

TINU syndrome is a rare disease that predominately affects young women and children and is defined by the presence of tubular interstitial nephritis and uveitis [3]. The diagnosis of TINU syndrome is characterized by the presence of uveitis and interstitial nephritis in the absence of a secondary cause of either condition. The etiology is unknown; however, it is thought to be an immune-mediated process in genetically susceptible individuals [4] that is thought to be provoked by antibiotics, non-steroidal anti-inflammatories [5], or infections. Moreover, it is frequently reported with other autoimmune diseases [6]. Evidence also suggests that the pathogenesis of TINU may be linked to modified C-reactive protein (mCRP), a common autoantigen in the uvea and tubular cells [7]. Various systemic diseases that have renal and ocular manifestations need to be considered as differential diagnoses. Diseases that can present with ocular abnormalities and acute interstitial nephritis include sarcoidosis, Sjögren's disease, tuberculosis, and Behçet syndrome. Many of these disorders present with multi-organ involvement as well as additional ocular manifestations that are distinct from uveitis. Currently, there are no standardized treatment guidelines available to manage this condition, but corticosteroids are used as first-line therapy [8]. In this case report, the patient had a history of CMV and presented with several weeks of flu-like symptoms; however, it cannot be definitively determined whether a possible viral infection triggered the TINU syndrome or if symptomatology is a consequence of the TINU syndrome itself. One limitation of this case report is the patient received three days of amoxicillin that was prescribed by her primary care physician which is known to cause drug-induced interstitial nephritis. Clinical findings in drug-induced acute interstitial nephritis (AIN) are related to allergic-type reactions with symptoms of low-grade fevers, maculopapular rash, and peripheral eosinophilia within a few days of initiation of the offending drug [9]. Even though biopsy findings are consistent with antibiotic-induced AIN, the absence of other clinical findings makes this diagnosis unlikely.

The findings of tubulointerstitial nephritis (TIN) on histopathology include tubulointerstitial cell infiltrate, predominately consisting of lymphocytes and monocytes, with the presence of few neutrophils, plasma cells, and eosinophils. The glomeruli and blood vessels are generally preserved [10]. The kidney biopsy in this patient revealed predominant interstitial cell infiltrates consisting mainly of mononuclear cells with few eosinophils and plasma cells but focal involvement of the proximal tubules with neutrophils and diffuse thinning of the glomerular basement membrane. The higher density of neutrophils was suggestive of bacterial infection [10]. However, further investigative studies involving urinalysis, urine culture, and CT imaging did not reveal an infectious process. There are no cases in the literature showing a relationship between TINU and diffuse thinning of the glomerular basement membrane.

The first diagnostic criteria were developed by Mandeville et al. [11]. The diagnosis of TINU can be made when uveitis is diagnosed within two months or 12 months after the onset of histopathological confirmation or diagnosed AIN, while other systemic diseases to cause AIN or uveitis have been excluded [11]. The patient had bilateral uveitis diagnosed within two months of biopsy-proven AIN fulfilling the diagnostic criteria.

The long-term prognosis of TINU is variable with incomplete recovery and persistent chronic kidney disease in certain cases. As with other rare syndromes, there are limited evidence-based treatment guidelines for TINU. Systemic glucocorticoid therapy such as prednisone in the 1 to 1.5 mg/kg/dose tapered over a few months has been used with favorable outcomes. However, the duration and the tapering schedule should be adjusted to how well the patient responds to treatment [8]. One study involving a one-year follow-up of 10 patients with TINU suggested that patients had better long-term renal versus ocular prognosis, as uveitis had frequent relapses despite glucocorticoid treatment [12]. When uveitis relapses prove to be treatment resistant, the introduction of immunosuppressive agents such as methotrexate, azathioprine, or cyclosporine has been shown to improve intraocular inflammation and prevent relapses [13]. In this case, the patient’s renal function normalized after a prolonged steroid taper, but the uveitis recurred necessitating steroid-sparing therapies such as methotrexate, mycophenolate, mofetil, and now adalimumab infusion.

## Conclusions

This case study on a 29-year-old female who presented with uveitis and tubulointerstitial nephritis highlights the high degree of clinical suspicion required to diagnose TINU. There are no treatment guidelines; however, glucocorticoids have been proven to be efficacious; however, the dosage and duration of therapy need further investigative studies.

## References

[1] Dobrin RS, Vernier RL, Fish AL (1975). Acute eosinophilic interstitial nephritis and renal failure with bone marrow-lymph node granulomas and anterior uveitis. Am J Med.

[2] Li C, Su T, Chu R, Li X, Yang L (2014). Tubulointerstitial nephritis with uveitis in Chinese adults. Clin J Am Soc Nephrol.

[3] Abed L, Merouani A, Haddad E, Benoit G, Oligny LL, Sartelet H (2008). Presence of autoantibodies against tubular and uveal cells in a patient with tubulointerstitial nephritis and uveitis (TINU) syndrome. Nephrol Dial Transplant.

[4] Levinson RD, Park MS, Rikkers SM (2003). Strong associations between specific HLA-DQ and HLA-DR alleles and the tubulointerstitial nephritis and uveitis syndrome. Invest Ophthalmol Vis Sci.

[5] Moledina DG, Perazella MA (2017). Drug-induced acute interstitial nephritis. Clin J Am Soc Nephrol.

[6] Tan Y, Yu F, Zhao M (2011). Autoimmunity of patients with TINU syndrome. Hong Kong J Nephrol.

[7] Tan Y, Yu F, Qu Z (2011). Modified C-reactive protein might be a target autoantigen of TINU syndrome. Clin J Am Soc Nephrol.

[8] Clive DM, Vanguri VK (2018). The syndrome of tubulointerstitial nephritis with uveitis (TINU). Am J Kidney Dis.

[9] Praga M, González E (2010). Acute interstitial nephritis. Kidney Int.

[10] Joyce E, Glasner P, Ranganathan S, Swiatecka-Urban A (2017). Tubulointerstitial nephritis: diagnosis, treatment, and monitoring. Pediatr Nephrol.

[11] Mandeville JT, Levinson RD, Holland GN (2001). The tubulointerstitial nephritis and uveitis syndrome. Surv Ophthalmol.

[12] Takemura T, Okada M, Hino S (1999). Course and outcome of tubulointerstitial nephritis and uveitis syndrome. Am J Kidney Dis.

[13] Gion N, Stavrou P, Foster CS (2000). Immunomodulatory therapy for chronic tubulointerstitial nephritis-associated uveitis. Am J Ophthalmol.

